# Sharing Perceptual Experiences through Language

**DOI:** 10.3390/jintelligence11070129

**Published:** 2023-06-26

**Authors:** Rosario Caballero, Carita Paradis

**Affiliations:** 1 Facultad de Letras, Universidad de Castilla-La Mancha, 13071 Ciudad Real, Spain; mrosario.caballero@uclm.es; 2 Centre for Languages and Literature, Lund University, 22100 Lund, Sweden

**Keywords:** architecture, built space, vision, hearing, smell, taste, touch, motion

## Abstract

The aim of this article is to shed light on how sensory perceptions are communicated through authentic language. What are the language resources available to match multimodal perceptions, and how do we use them in real communication? We discuss insights from previous work on the topic of the interaction of perception, cognition, and language and explain how language users recontextualise perception in communication about sensory experiences. Within the framework of cognitive semantics, we show that the complexities of multimodal perception are clearly reflected in the multifunctional use of words to convey meanings and feelings. To showcase the language resources employed, we base our findings on research on how architects convey their perceptions of built space. Two main patterns emerge: they use multimodal expressions (*soft*, *bland*, and *jarring*) and descriptions of built space through motion (*the building reaches out*, or routes and directions such as *destination*, *promenade*, *route*, or *landscape* in combination with verbs such as *start* and *lead*) in which case the architect may either be the observer or the emerged actor. The important take-home message is that there is no neat and clear a priori link between words and meanings, but rather “unforeseen” patterns surface in natural production data describing sensory perceptions.

## 1. Introduction

In his book, *The senses considered as perceptual systems*, James J. Gibson (1966) presents his radical, pioneering ideas about the senses and perceptual systems. He rejected the current mechanistic stimulus-response formula and established the foundation of ecological psychology as a field. His ideas are nicely illustrated in his example of a fire.

*[A fire is] a terrestrial event with flames and fuel. It is a source of four kinds of stimulation, since it gives off sound, odor, heat and light […] One can hear it, smell it, feel it, and see it, or get any combination of these detections, and thereby perceive a fire. […] For this event, the four kinds of stimulus information and the four perceptual systems are equivalent. If the perception of fire were a compound of separate sensations of sound, smell, warmth and color, they would have had to be associated in past experience in order to explain how any one of them could evoke memories of all the others.*

(Gibson 1966, pp. 54–55)

The issue of ecological science is very topical in contemporary research, not only in psychology but also in neighbouring fields such as in those language science approaches that see language and cognition as embodied and grounded in perception. The question, then, is how is it possible for human beings to arrange everything in manageable forms for it not to become “one great blooming, buzzing confusion” (James 1890, p. 462). This becomes more complex if we consider that it does not only concern perception but also cognition and communication through language. Even today we know very little about how these systems interact with one another, and research on how sensory experiences and meanings are conveyed in authentic communication is scarce, even though much of the current research operates on the basis that cognition and communication are largely shaped by our sensorimotor and perceptual systems and by our bodily interactions with the world (e.g., Barsalou 2020; Connell and Lynott 2010; Lakoff and Johnson 1980, 1999; Louwerse and Connell 2011; Pecher et al. 2003, 2004; Spence 2020; Tomasello et al. 2018; Turatto et al. 2004; Pecher and Zwaan 2005; Shapiro and Spaulding 2021; Zwaan 2014).

Providing a review of previous work on sensory–motor perceptions in the language sciences and beyond, we explore how those experiences are shared in human communication and then take a special look at architectural communication. We synthesize findings based on our research during more than two decades on how architects experience space through the lens of how they communicate their experiences and thinking through language. Our choice of topic is motivated by the issues raised by Gibson above, namely, how are the compounds of experiences afforded by space described by architects? We aim at showing in what way their descriptions reflect the perceptual experiences in terms of the language resources at their disposal. The domain of architecture is appealing since it is commonly considered to be a spatio-visual profession—a view that architects, in general, are not entirely satisfied with. While admitting that vision is a critical sense in their profession, they also insist that architecture has a firm, highly multi- and intermodal grounding in sensorimotor experiences and the perceptual system. Being aware of the visual bias towards built space, the Royal Academy of Arts in London organized an exhibition called “Sensing Spaces. Architecture Reimagined” in 2014, an event launched with the aim of making visitors think about their experiences with built space. Consider the introduction to the leaflet given to visitors:

*How does the room you’re sitting in make you feel? What is it about the soaring roof of a railway station, the damp odour of a cellar, the feel of worn stone steps beneath your feet, the muffled echo of a cloister or the cosy familiarity of your lounge that elicits glee, misery, fear or contentment? We’ve tasked seven architects with reawakening our visitors’ sensibilities to the spaces around them—bringing to the fore the experiential qualities of architecture*.^1^

The role of sensory perceptions in people’s understanding of space is stressed throughout the official guide to the exhibition, where the organizers quote Royal academician Colin St John Wilson’s claim that “knowing how to ‘see space’, or how to be spatially attuned, is an ability with which we are all born”, and this knowing how to “see space” involves “a wide range of sensual and spatial experiences” such as “roughness and smoothness, warmth and cold, being above or below, inside, outside, in between, exposed or enveloped.” If, as Wilson and many others claim, architecture must be *felt* to be *understood*, our exploration of how architects make use of language to talk about built spaces may serve as a suitable domain. Another motivation for selecting architecture and how architects communicate their work is that we are thereby able to highlight the challenge of expressing meanings about sensory perception in communication about built space. Architects are obliged to talk about their ideas and their conceptualisations of space not only to other architects but also to people outside the field. In other words, architects are language users like everybody else and, hence, our exploration of the language of architects can be extended to describe and theorize about word meanings in language more generally.

Our discussion is organized as follows. In Section 2, we give an account of the role of sensory perceptions in the language sciences and the approach known as embodied cognition. Section 3 is devoted to discussing how architects use language to describe built spaces, particularly the way sensory experiences in those spaces are communicated. This section is divided into three parts: the first gives a summary of the production data we used for our case study of architectural communication. The next section deals with descriptions related to how language resources are employed to describe sensory properties of built space, and the third focuses on aspects related to motion and built space. Section 4 concludes the article.

## 2. The Advent of Embodied Cognition

In the latter part of the 1900s, the received theory of meaning in the language sciences was the amodal symbolic approach based on logic, formal semantics, and computer science (for general outlines of the main theoretical developments, see Gärdenfors 2000, pp. 33–56; Geeraerts 2010). Amodal approaches to conceptual knowledge hold that knowledge representations are abstract symbols that are completely disconnected from systems of sensory perception and motor action. This means that understanding the meaning of an expression in language only requires access to the abstract symbolic system, and how those symbols can be manipulated by explicit rules to form meaning in language (Fodor 1975). There were also structuralist approaches to meaning in language that argued that meanings are not substantial, but meanings of words are in the relations between words (Lyons 1977).

Both of those approaches remain but have, in many areas of semantics, been usurped by the embodied cognition approach to meaning in language. This theoretical conception is the foundation on which the broad framework of cognitive linguistics is based. It rejects amodal, disembodied views and instead embraces the idea that the pairing of language and cognition is grounded in patterns of our bodily constitution and our perception of the world (Talmy 2000, 2003; Tomasello 2008). Cognitive linguistics is an approach that sees human thinking as ultimately motivated by our corporeal experiences in and with the world, and this is reflected in the way we use language to communicate those experiences. These ideas have not only attracted a lot of theoretical attention, but they have also resulted in a fair amount of empirical work in philosophy, linguistics, psychology, and cognitive science (e.g., Barsalou 2020; Beveridge and Pickering 2013; Bianchi et al. 2017; Borghi and Cimatti 2010; Caballero and Paradis 2015, 2020; Damasio 1989; Gärdenfors 2018; Gibbs 2006, 2011; Goldberg et al. 2006; Hartman and Paradis 2021; Kemmerer 2015; Pecher and Zwaan 2005; Lakoff and Johnson 1980, 1999; Paradis et al. 2013; Speed and Majid 2020; Varela et al. 2017).

Central to the cognitive linguistics school of thought is the idea that language is a structured collection of meaningful categories or lexical concepts, which are formed based on our experiences in the world. The way we perceive and understand the world around us is the way we express ourselves and vice versa. Categories help language users to manage knowledge in a way that reflects the needs of human beings, and research based on comparisons across languages demonstrates that there are both commonalities and differences between languages (e.g., Geeraerts 2018; Jędrzejowski and Staniewski 2021; Koptjevskaja-Tamm and Nikolaev 2021; Koptjevskaja-Tamm 2022). This means that meanings are to some extent subjective, view-pointed, and sensitive to individual, situational, and linguistic contexts. Yet not all aspects of conceptual knowledge are of equal weight in meaning making. Only parts of a knowledge base that are relevant to the contextual situation and to the interlocutors involved need to be activated and made salient (Langacker 1987, pp. 158–61; Paradis 2015a). For instance, the spatio-visual representation we have of an orange is that it is roundish and its colour is orange. We recognize the texture or the peel and the feeling of eating an orange, its sweet smell and sweet–sour taste. We know that they are a type of fruit that grows on trees and can be used in a wide range of dishes or eaten fresh. These factors all represent core meaning aspects shared by most speakers in our culture. Those qualities are generic and characteristic, while there is also idiosyncratic knowledge such as Sheila’s father used to grow oranges in his garden and her sister loved oranges. Those are peripheral aspects at the outskirts of the range of centrality factors lacking in the conventionalized form-meaning coupling of *orange*/orange. They are peripheral and private and not intrinsic to the representation of the entity, while all the perceptual aspects are central to the notion of orange. However, not all of them need to be always made salient in communication.

Despite the foundational importance of perceptual experiences and our bodily configuration, it is fair to say that cognition has been given the better part in explanations of how we construe meanings in most work in the language sciences (but see the work on the role of the perceptual system in meaning making by Caballero et al. 2019; Hartman and Paradis 2021; Paradis 2015b). This situation calls for more attention to the foundational idea that the two sides of expressions, i.e., language form and meaning/cognition, are experientially grounded and constrained by our physiological, sensory–motor constitution, which constitutes the prerequisite and the constraints of our use of language, as nicely put by Shapiro and Spaulding (2021).

*A lake thought re-activates areas of visual cortex that respond to visual information corresponding to lakes; areas of auditory cortex that respond to auditory information corresponding to lakes; areas of motor cortex that correspond to actions typically associated with lakes (although this activation is suppressed so that it does not lead to actual motion), and so on. The result is a lake concept that reflects the kinds of sensory and motor activities that are unique to human bodies and sensory systems. Lake means something like “thing that looks like this, sounds like this, smells like this, allows me to swim within it like this.”*

Indeed, a substantial body of research on sensory meanings suggests that cognition and language are structured in a cross-modal way, which, in turn, is a consequence of the fact that the world, to a substantial degree, is perceived in a cross-modal way that includes the entire sensory–motor system (Caballero and Paradis 2015; Gibson 1966; Sathian and Ramachandran 2020; Winter 2019). However, much more research on the role of perceptual experiences and language is needed. It is true that some research has currently been carried out with the use of a wide range of empirical methods ranging from data-driven statistical methods (e.g., Hörberg et al. 2020), discourse analytical and semantic corpus methods (Caballero et al. 2019; Caballero and Paradis 2020), behavioural experiments of different kinds (Bianchi et al. 2011, 2013, 2017; Lynott et al. 2020; Paradis et al. 2009) and brain research (e.g., Hörberg et al. 2020; Olofsson et al. 2020; Pulvermüller 2005). A good deal of this research also includes combinations of methods (e.g., Paradis and Willners 2011; van de Weijer et al. 2014).

An important insight from research in language, cognition, and perception is that words do not *have* a set meaning but *evoke* meanings when they are used in human communication (Paradis 2015a). For instance, *soft* is a form that ranges over all five basic sensory domains, vision, hearing, smell, taste, and touch, as in *soft colours*, *soft music, soft smell of elder, soft taste of vanilla,* and *soft texture of cotton wool* (Lynott et al. 2020; Paradis 2015b), and beyond these meanings *soft* may also be used to describe human personality, *a softhearted person*. Moreover, observations have been made about descriptions of audition where motion is recruited to describe the perceptual experiences in a soundscape such as the intertwining of sound and motion in *he slammed his way through the door* and *the door buzzed open* (Caballero and Paradis 2020) and motion in descriptions of space as in examples such as *the trail climbs 1000 meters* and *roads or the mountain range go from Mexico to Canada* (Matlock 2010; Matlock and Bergmann 2015; Talmy 1983, 2000).^2^ This flexibility of meaning application in language use is what we expand on in this article with a focus on spatial descriptions by architects. What is built space for them?

A topic closely related to this flexibility of meaning related to sensory expression such as *soft* is that they may form antonymic relations of opposition along the dimensions that connect them, such as *soft–hard* (touch, vision, and hearing), *soft–bright* (vision and hearing), *soft–sonorous* (hearing), *soft–grainy* (touch and vision), and *soft–bitter* (taste). Construals of antonymy of individual adjectives constrain their meaning application in one or another sensory domain in a systematic way (van de Weijer et al. 2023). For smell, for instance, *soft* and *hard* would not be a good pair of antonyms, but *soft–sharp* would. In previous approaches alluded to at the beginning of this section, the researchers’ focus was either on setting up such pairings as idealized models of language structures without paying attention to speakers’ behaviours and language use (e.g., Lyons 1977) or with a focus on formalization of a range of predetermined words and the analysts’ interpretations of them (e.g., Kennedy and McNally 2005). In contrast, what approaches to language in the cognitive framework contribute is that pairings of forms and meanings are dynamic and sensitive to context. When two lexical items are used to express opposition in discourse, they become firmly instantiated in a given conceptual dimension (Paradis and Willners 2011). 

Finally, yet another type of research that is important for insights about form–meaning flexibility that has been carried out is work using large-scale statistical elicitation techniques of participants’ assessments of individual words in isolation, and their meaning potentials across the senses and their assessments of the proportionality of the sensory modalities in the meanings of each word. Lynott and Connell (2009, 2013) measured speakers’ perceptions of sensory meaning ranges of words (adjectives and nouns) that might be associated with one or more interpretations, e.g., *thin, yellow, glowing,* and *dark,* and calculated the patterns of the strength of association to the five sense modalities. The participants were asked to indicate to what extent they experienced something to be, say, *thin* “by feeling through touch”, “hearing”, “seeing”, “smelling”, and “tasting” on a scale from 0 (not at all) to 5 (greatly). They showed that most of the words tested (423 adjectives and 400 nouns) were associated with more than one sense modality. There was also a follow-up on this technique in a much larger study, *The Lancaster Sensorimotor norms*, where many more words were added, as well as interoception and motor meanings (Lynott et al. 2020). They made use of Amazon’s Mechanical Turk platform to measure the strength of 39,707 English lemmas. These norming studies are valuable resources for work on knowledge representation in different fields and for conceptual representations for the language sciences as is research on patterns of language use across different contexts and genres (Louwerse 2018; Caballero and Paradis 2018, 2020).

With this description of previous work on perception, cognition, and language, we now proceed to investigate how this pans out in architects’ production data, namely, in how architects describe built space to their interlocutors.

## 3. Explaining Built Space

In this section, we describe the ways in which architects use language to share the experiences afforded by built spaces. This is a challenging endeavour in that, although explaining what buildings (and other artefacts such as sculptures or paintings) look like appears to be reasonably straightforward, describing what they smell or feel like might be a more challenging undertaking. We start by describing the data sources on which our account is based and then proceed to take a close look at how architects conceive of sensory–motor properties of built space through the lens of how they communicate their perceptions to others. With the goal of highlighting how perceptions are recontextualised and shared by architects, our analysis focuses on the semantics of examples from data sets compiled for different projects over two decades as described in the next section.

### 3.1. Data

The data used in this article are of two types. On the one hand, unless otherwise indicated, we make use of a corpus of 150 texts (120,000 words) retrieved from print magazines written by and for architects and enjoying a high status within the community (*Architectural Record*, *The Architectural Review*, *Architectural Design*, *Architecture*, *Architecture Australia*, and *Architecture SOUTH*) and architecture websites such as arcspace.com, archdaily.com, architizer.com, and architectmagazine.com (for more information, see Caballero 2006, 2009, 2017, 2020). On the other hand, we also use data from a project conducted by architect Hernan Casakin (Ariel University, Israel) with 60 MA students of TU Delft where Rosario Caballero collaborated in the analysis and classification of the metaphorical language used by the students in the task. These were organized into twenty teams of three members each and the teams were asked to redesign the entrance area of the faculty to make it more enjoyable. Starting from the assumption that architecture students would be visually biased, the groups were given both textual and visual stimuli to see which of these was more helpful in the generation of design ideas. All the sessions were filmed and recorded. The students were asked to discuss their work aloud as they drew the sketches and to provide a short report at the end of the task (for a detailed discussion, see Casakin 2019). The oral data used in Section 3.2. belong to the transcriptions of those sessions.

### 3.2. Word Meanings within and across Sense Modalities

Architects are often considered visual thinkers given the weight of drawing in their job and the role of images (pictures, 3D models, etc.) in the design process, and the importance of the final appearance of buildings. However, as has already been hinted at, their language is not exclusively instantiated in meanings of words of vision. Consider for instance, “the Sonoran desert around Tucson is visually **fragile**—easily thrown into imbalance by a **jarring** building”, where the tactile descriptor, *fragile*, and an aural descriptor, *jarring*, in this context are not about touch or sound but about visual properties of the space, as clearly indicated by *visually*. Inasmuch as lay people hear, smell, and feel the various spaces involved in their daily routines, public as well as private, architects also say that their work is, indeed, multisensory/multimodal, and that vision engages the other senses as well (Rasmussen 1959; Bloomer and Moore 1977; Pallasmaa [1996] 2005; Zumthor [1998] 2006; Seamon 2007). Indeed, architectural descriptions make use of expressions such as *craggy*, *fluid*, *enveloping*, *clammy*, *loud*, *warm*, *bland*, *stuffy,* or *crisp* in order to communicate the sensory properties of buildings, i.e., to express what buildings feel like in a form that their users can understand and, ideally, also relate to through their senses (Caballero and Paradis 2013; Caballero 2009, 2017, 2020).

The most conspicuous case of cross-modal expressions in architecture is the use of primarily aural descriptors to portray visual experiences, as illustrated by the expressions in bold in (1)–(3).

1. Juxtapositions of sleek finishes [inside the building] such as citrus-colored partitions and tiny halogen spotlights feel **cacophonous** against the rough timber walls and columns.^3^
2. Tucked behind two refurbished cottages that now serve as flexible office and guest quarters, [the house] is a **cacophony** of pitched roofs, steel awnings and **crisp** eyelid-like window hoods.
3. [Chief architect] recalls, “They said, ‘Maybe this building could be a little **quieter**.’” [And they] set to work defining just what “**quiet**” could mean, esthetically speaking. “We talked a lot about trying […] to make it a **quiet** place.” That discussion soon led to ideas of garden, the metaphor that began to inform their design studies. […] nonliteral notions of garden did more to germinate this inventive building’s abstract qualities as a salve for the **sensory whipping** delivered by its suburban context. […] The building’s main event is inside […] the architects created an alternating **rhythm** of angled surfaces to bounce light and disperse sound. […] the sparsely landscaped lawn was […] a way to buffer the **noise**, both aural and visual, that is certain to kick in when the adjacent corner lot becomes a gas station or convenience store.

The reverse is also true, namely, the use of visual and tactile descriptors to refer to sound, as shown in examples (4)–(6).

4. Since concert halls are large open spaces, they present opportunities for **sound abrasions** and **acoustic glare**.
5. The zigzag channels are made from aluminum that is perforated to achieve **acoustic transparency** […] the most unusual feature [is] as a set of velour curtains that hang between **the acoustically transparent skin** of the auditorium and the concrete outer wall.
6. **Acoustic shadow** created by Podium Building; as a result of the angle created between the source and podium edge, a portion of the facade is **acoustically shaded**.

Examples (4) and (5) evoke the representations of aural phenomena critical in the design of buildings devoted to performances such as theatres and concert halls through visual and tactile expressions. *Glare* is used to evoke the harsh quality of sound inside a building caused by walls or surfaces that are too flat and/or too smooth and *transparency* is the ability of sound waves to pass through certain materials and be absorbed in space rather than bounce off or echo in it. In (4), *abrasion* from the domain to touch is used to refer to aural effects in buildings. Finally, example (6) describes sound effects in terms of visual *shadow* and *shade*.

**Figure 1:**
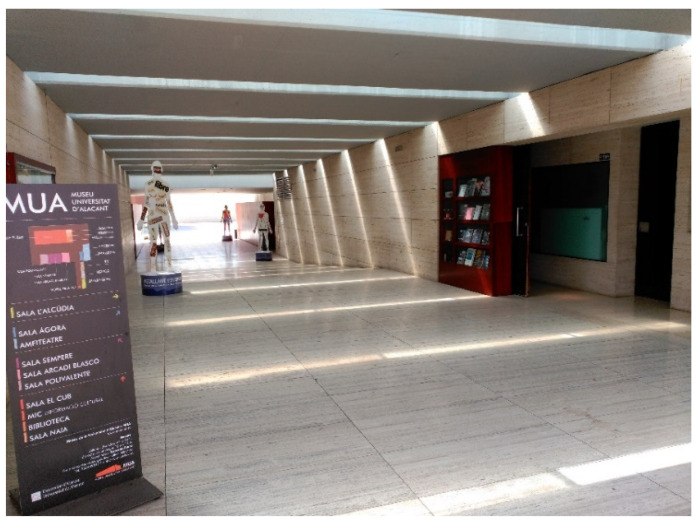
Museum of the University of Alicante (MUA). Photograph property of the authors.

From a language science point of view, the highly important take-home message of the various descriptions of the perceptual affordances of the sensescapes of built space and their effects on human engagement with them is the dynamic cross-modal descriptions. The examples put the spotlight on the high degree of malleability in word meanings in different contexts in authentic communication, in both the polysemous cross-modal use of form and meaning in communication as well as the use potential that expressions in language evoke more than one interpretation at the same time. This is evident in *cacophonous* in (1), which describes both the visual *and* textural properties of an interior space, and *quiet*, *rhythm,* and *noise* in (3), which evoke both aural and visual information. In fact, an architect would argue that *rhythm* also conveys textural properties inherent in the manipulation of light by means of structural and ornamental elements, as apparent in Figure 1, showing a photo of the Museum of the University of Alicante (MUA).

In other words, although mainly concerned with sight, word meanings from the domain of sound are also used to communicate spatial properties accessed through touch. This is because spatial sequences and patterns such as spatial *rhythm* or *choreography* ultimately endow built spaces with a textural or tactile “feel”, even if such patterns are first accessed through our eyes. Thus, the use of aural language by architects is clearly cross-modal in that word meanings, typically of sound experiences, convey meaning referring to the sight and touch of built spaces to provoke the perception of hearing, sight, and touch. In fact, in architecture, the notion of texture covers both the tactile and visual quality of the surfaces of buildings, as explained by Roth and Rasmussen in their lecture notes, “Texture and Light in Architecture”, for the Introduction to Architecture course offered at Çankaya University.

7. Texture has various meanings […] Optical texture could be given by the organization of architectural elements, such as windows, doors, solids or voids. The repetition of elements creates a pattern that is observed as an optical texture. Tactile texture on the other hand could be given by building materials, such as concrete, brick, stone, glass, steel etc. [You can achieve both, as] In Baker House, in addition to a visual rhythm, Alvar Alto has used rough clinker brick to be able to give the building a tactile texture. Moreover, he had the bricks laid in a random pattern to add visual texture. [www.archplea121.cankaya.edu.tr] (accessed on 1 January 2023)

Accordingly, students are trained from the very beginning to pay attention to texture in their projects, as shown in examples (8) and (9) where MA students of architecture and urbanism discuss space in the preliminary stages of an assigned project.

8. S1: Yes and also with this [taps image provided a visual stimulus] … with this thing is the idea of … of a **soft** and **hard** places.
  S2: yes it’s like **soft** this is more of **soft** [points to sketch they’re drawing]
  S1: yeah there you have more **hard** and here have maybe this …
  S3: and why would we **split it up** in **soft** and **hard**…it could be **scattered around**?
  S1: yeah yeah … like the main [inaudible] so this one would be **hard** anyway otherwise…
9. [student takes visual stimulus] four was interesting ‘cause it seem like, er, this contrast between very **dense**, this **compact cluster** [draws a cluster plus some scattered dots], but then it’s like a very contrast with this **very open area**, so this show contrast between **density** and then **open** [inaudible] which is more **expansive**

What is interesting here is that the students make use of property descriptors such as *soft*, *hard*, *dense*, and *compact* which also evoke touch in their visual descriptions of space. The visual meanings are invoked by the arrangement of spaces, portrayed in a way that suggests that space is a malleable entity susceptible to being manipulated in various ways (see Caballero 2006, 2009, 2017 in this respect).

So far, we have shown how meanings of words are employed to describe various spatial aspects in architecture with examples retrieved from different communicative contexts in architectural discourse (architectural reviews, lecture notes, and students’ interactions). Some such terms are grouped in Table 1, where they are sorted according to the main source and target domains involved in their use in the data.

Table 1 offers a summary of cross-modal use and thereby points to the dynamics of meaning making in authentic production data. The table also highlights the importance of meanings other than vision, both for the design and experience of built spaces. Indeed, as pointed out already by both James (1890) and Gibson (1966), our experiences of the world are not monomodal, i.e., our senses do not work in an isolated, discrete manner but are highly interactive. In the case of architecture, where vision is critical, other sensory experiences also play important roles, as explained in a blog from Virginia Tech by Kristen Long.

10. Texture can make or break a structure or building when it comes to design. It can be a crucial part or architecture, creating pattern or rhythm and allowing the viewer to believe the piece moves through space. Textures create a different experience; they allow more than one sense to be used at once by just “seeing” it. Textures allow viewers see the building as well as imagine how it would feel.
  [https://blogs.lt.vt.edu/kristen3/2013/02/08/texture-in-architecture] (accessed on 1 January 2023)

While highlighting the multimodal quality of spatial experiences, Long also brings up one of the most recurrent experiential domains used by architects in their work, namely, motion. Motion is a complex, multisensory domain already noted by the classics (e.g., Aristotle and the scholastics after him). It was regarded as a *common sensible* or primary quality whereby humans deal with complex data by combining sensory and cognitive processes/activity.^4^ This dynamic, interactive approach to architecture is characterized by Yudell (1977, p. 59), who claims that “all architecture functions as a potential stimulus for movement, real or imagined”, a statement motivating his belief that basic architectural experiences have a verb form. In the next section, we discuss the use of motion to describe built space.

### 3.3. Fictive Motion and Built Spaces

As accounted for elsewhere (Caballero 2009, 2017, 2020; Caballero and Paradis 2013, 2015), architects often present built space in dynamic terms, i.e., as if moving in various ways which, of course, goes against the very essence of architecture, i.e., stability and immobility. Consider the following review of the national museum of contemporary art (Kiasma) in Helsinki.

11. The […] volume becomes the dominant form **reaching out to** the natural landscape. […] to the west are the information/ticket desk, the museum shop and a cafe which **opens out onto** a public terrace and the reflecting pool. A steel framed glazed canopy **extends out** from the vertical fissure […] A ramp **climbs up** the curved east wall of the void, **arriving** at the critical crossing point […]. Suites of enfilade double-height galleries **step up** the building in four split levels […] The underlying order of the building cannot be understood from a single vantage point, but **unfolds** cinematically as you move through a **landscape** of interior space. This is an architecture of **promenade**, yet without a prescribed or privileged **route**. Multiple lifts, stairs and ramps combine with the split-level galleries to create many possible **itineraries**. Passage between rooms occurs in a zigzag **trajectory** […]. **Circulation** always **returns** to the central orienting void. […] the wall also **rotates** from a 9.5-degree outward **tilt** at its southern end […]. The north end of the building **twists towards** the west […] Much of the daylight in the building […] is diffused by translucent glass which both intensifies the weak Nordic light and imparts a sense of quiet abstraction and detachment from the life of the city. So movement through the building becomes an introverted **journey**.

Here the building and some of their elements are described as *reaching out*, *climbing*, *stepping*, or *twisting* in their sites, and as having *itineraries*, *promenades*, or *routes* inside. The use of meanings of motions to depict static entities is variously known as *fictive motion* (Talmy 1988, 1996, 2000; Matlock 2004, 2017), *abstract,* or *subjective motion* (Langacker 1986, 2000; Matsumoto 1996) or, more comprehensively, *non-actual motion* (Zlatev et al. 2010; Blomberg and Zlatev 2014), and is often found in descriptions of household things such as cables or hoses and also of structures designed for human motion, whether this requires a vehicle (e.g., roads) or our own bodies (e.g., buildings). In (11), this dynamic portrayal is achieved through verbs such as *reach out*, *open out*, *extend*, *climb*, *arrive*, *step up*, *unfold*, *rotate*, and *twist*, which describe what those spaces look like. The descriptions are concerned with their physical properties through sight. The variety of motion meanings involved in the descriptions also suggests that choice of verb meanings is determined by the characteristics of the space itself. Meanings of words such as *reach out* and *extend* describe long, horizontal spaces, while verticality is depicted by meanings such as *climb* or *step up*. Consider, for instance, the captions of some of the images in a review of Massimiliano Fuksas’ Maison des Arts in Bordeaux.

12. The massive warehouse **runs along** the north side of site.
13. The roof **cantilevers towards** the street through two traffic lights.
14. The green prism **crouching**.

While all three expressions of motion are concerned with the external appearance of the building, *run* emphasizes the building’s length in combination with the absence of obstacles, which implies a reference to spatial fluidity, *cantilever* profiles the horizontal projection of a beam or cantilever in the roof of the building, and *crouch* emphasizes the size and/or bulk of the building.

Word meanings instantiated in the domain of motion in spatial descriptions invoke dynamism, innovation, and graphicness, while verb meanings such as *lie*, *sit*, *rest*, *stand,* or *rise* are static and passive. Many of the verbs used as descriptors come with a flavour of human activity and personification of built space. Architectural descriptions often offer depictions with properties of liveliness such as *hunker*, *ease*, *sweep*, *sprawl*, *inch out*, *clamber*, or *unfurl* and thereby provide an even more dynamic portrayal. Examples (15)–(17) show how some such verbs are used.

15. The garden, which **rambles into** the house through a number of small courtyards, is an apt reminder of how a home should be occupied.
16. The interior open library **wraps around** staff spaces, a large community room, small meeting spaces and building services.
17. This sumptuously landscaped park in Santa Monica includes […] ramps that **clamber over** a drought-defying fountain, and […].

The most extreme case in this respect involves action verbs such as *cantilever* above and *rake*, *bunch*, *ramp*, *cascade*, *scissor*, *funnel*, *fan*, or *corbel*, to mention but a few of them found in architectural texts. By way of illustration, consider examples (18)–(21).

18. Customers descend to the store from the parking levels by elevators or by stairs that **scissor down** through the three-story space.
19. At north and south ends are stands for hardier (and poorer) fans, unshaded and **raking up** at a steep angle.
20. Here, the walls **curve** gently **backwards** until they get to the seventh floor, where they **crank** quite severely **back** to obey planning profile rules.
21. The driveway **slips in** under the west courtyard wall and then **ramps up** steeply to the entrance level.

In cases such as these, particles such as *down*, *up,* or *in* express direction of movement, which add shape (topology) to the descriptions through the verbs of action, e.g., *scissor*, *rake,* or *crank*. In other words, while the particles *backwards* and *back* express the direction of motion in (20), *curve* and *crank* specify the shape of the walls. The fact that architecture students often replicate such uses further points to their ubiquity in architectural communication, as shown in these examples from the Delft experiment.

22. I think you should combine these two into one design because it’s also the same shape like it **starts** very small and then **curves around** …
23. so [starts drawing boxy things] what I thought is sort of a box was **coming out** of the plinths, it was **sticking through**, the entry is maybe is here, and in the box there is the canteen...
24. so I kept this as the entrance to the building and **sloping out** [students start a discussion on entrances to the building].

The uses discussed so far suggest a visual metaphor that may be formalized as form is motion whereby particular layouts or appearances of form (the metaphorical targets) are seen as reminiscent of the kind of movement encapsulated in the motion meanings of the verbs (the metaphorical sources). Put differently, the description of spatial arrangements and topologies draws upon our more basic understanding of particular ways of moving (for a thorough discussion, see Caballero 2009, 2017, 2020).

Another use of motion meanings points to the role of architecture as a stimulus for movement. This point was raised by Yudell and further described by well-known architects within the architectural canon such as Bloomer and Moore and Pallasmaa. Thus, Bloomer and Moore (1977, pp. 86–88) describe the temple complex at Monte Alban (Mexico) as follows.

*The temple complex […] seems to have been built around the act of climbing. There, thousands of feet above the valley floor, a flat plaza was made from which each temple was entered, up a flight of steps, then down, then up again higher to the special place. To arrive at the largest temple, one went up, then down, then up, then down, then farther up again. […] getting there is all the fun. (Italics in the original)*

A similar view underlies (Pallasmaa [1996] 2005) claim that architecture is best appreciated as we interact with it, i.e., as we move inside buildings, but he is more explicit about the embodied, cross-modal quality of the whole event:

*I confront the city with my body; my legs measure the length of the arcade and the width of the square; my gaze unconsciously projects my body onto the facade of the cathedral, where it roams over the mouldings and contours, sensing the size of recesses and projections; my body weight meets the mass of the cathedral door, and my hand grasps the door pull as I enter the dark void behind. I experience myself in the city, and the city exists through my embodied experience. The city and my body supplement and define each other. I dwell in the city and the city dwells in me.*

Similar aspects are revealed in the education guide for the Sensing Spaces exhibition, where some of the architects’ works are described as follows.

25. Chinese architect Li Xiaodong is not seen or experienced as an object in space. Instead, it builds upon the sequential experience of visiting the Academy—via courtyard, town-palace, grand staircase and Beaux-Arts gallery […] Experienced as a choreographed one-way **route**, the timber frame is […] An acrylic raised floor is illuminated by LEDs, and plywood-lined niches provide accents along the **route**, culminating in [what the architect likens to] a Zen garden […] presented as the final scene on **a route that the architect likens to ‘a walk through** a forest in the snow at night’. […] In this place, visitors are invited on **a journey of discovery** and to sense that **alternative worlds run alongside their path** and intersect with it. They can experience the different spaces, from the narrow passageways and intimate niches, to the expansive Zen garden. […] this is an unfolding story that is best moved through slowly and appreciated over time.

This explicit focus on dynamics and interaction is further reinforced by some of the questions included in the exhibition leaflet and education guide, which point to a change from an ocular-centric way of experiencing architecture to a more enactive, sensory one.

1. Describe what you experienced walking through this installation.
2. Did you feel differently in the passageways and niches?
3. How did you react when you emerged in the Zen Garden?

Such an approach asks for a broader use of language resources and a more inclusive theoretical framework that takes seriously authentic human communication as well as humans’ encounters with the world around them when describing built space. 

In descriptions of fictive motion, we do not only find motion in expressions conveyed by verbs, but we also find the abundant use of motion expressed by nouns. Some such nouns refer to sense spaces in terms of *circulation*, *promenades*, *routes*, *itineraries*, or *trajectories*, first illustrated in example (11) and present in (25) and are, of course, recurrently brought up by the Delft’s students when discussing their projects.

26. one of my first ideas and my favourite one was […] different, er, **paths**, so, actually, to make this as the **pathways** and then have **hills** in between them, so to create like a **hills landscape** that would define different spaces that would become much freer and more playful] and give opportunities for use.
27. ST 3 starts drawing on ST2’s sketch and says: what I got from this was something different actually, er, what I got was like this **centre point** also this sort of **axis** … that comes up and from here I got this **central axis**, **secondary axis** here … so it’s more like **directionality** with a **frame circulation** […].
28. ‘cause this is more a, a, a **route**, also from this side and, I don’t know, what I see here like city facilities but more, is more about the **route** and the, the **orientation** of the paths which are going into, er, which are letting you in the building”
29. ST1: “I really thought literally of the **rollercoaster** and saw this element going up and down and around.
30. S1: You said two things about **navigation** … I think that is **forced navigation**
  S3: if you have activities then that is the **circulation** that you want to reach” [3 clarifies the other idea and 1 agrees].
  S2: if you make a connection to the park and it’s good to put these trees also in this…**pathways** […] you have other ways of entering this public space […] you can also like dictate one major access that all the **flows** go through.

All the above examples illustrate an interactive scenario whereby moving inside a building is presented as an imaginary, virtual tour within and along its sense spaces, variously referred to as *paths*, *pathways*, *axes*, *routes*, and even *hills* (26), and where architects must ensure ease of *circulation* (27, 30), *orientation* (28), or *navigation* (30) for the building’s future users.

A final use of motion language in architectural discourse is more genre oriented, i.e., is typical of the texts known as architectural reviews and devoted to describing and assessing a noteworthy building, both in specialized magazines and good quality newspapers. In addition to discussing the aesthetic, technical, and constructive properties of architectural projects, a well-crafted review also attempts to translate a holistic experience of buildings into words, which often starts from an assessment of their visual properties to gradually guide the readers through their inner spaces, taking them on a tour that is often used as a blueprint for how to organize the commentary (Caballero and Paradis 2013). This is the case of a review of the Luxor theatre in Rotterdam, introduced as “a rich internal landscape of interacting layers that combines contingencies of site with the rituals of theatre going”, an experience described in the following terms:

31. Depositing their coats at the counter to the left, [people] **turn to start on** one of the three batteries of stairs **rising** to higher levels, **going to** left or right depending on **destination**. As they **rise** through the building, each new stair invites them to the next stage of the **promenade** until they find the appropriate level for their seat. Unprecedented is the delightful **sloping route** along the south edge on top of the lorry ramp, which is treated as **a series of very long steps** [...]. It **winds** irresistibly round, gathering more stair **connections** as it **goes**, and culminates in a double-level bar and restaurant [...]. Further stairs within this volume **lead to** an upper bar level and to a whole additional foyer **leading back** the other way to another bar above the entrance. The sequence of spaces–every bit a contrived **promenade architecturale**–is enriched by careful framing of views with various scales of window. Like the Philharmonic in Berlin [...] it provides a kind of **internal landscape** of interacting layers where the people of Rotterdam can parade in their finery to see and be seen, creating a theatre of the interval almost as important as that of the stage.

The passage features the two main types of fictive motion discussed in this article: firstly, we find the use of motion verbs such as *rise*, *go*, and *wind* to represent the illusion of movement created by the external appearance or arrangement of some building elements (e.g., stairs), and secondly, we find places and paths that in some way or another involve or entail motion such as *destination*, *promenade*, *route,* or *landscape* in combination with verbs such as *start* and *lead* to recreate some of the real motion experiences afforded to its future users. In other words, the text does not describe a scene, but an imaginary, prospective one where readers are offered a virtual tour of the Luxor theatre by means of motion language used to (a) describe the looks and function of the building’s various spaces, (b) help readers imagine what experiencing its spaces may actually feel like, and (c) act like some sort of blueprint for organizing the description in a coherent and engaging way.

## 4. Conclusions

The aim of this article was to expound on how language is used to share experiences of sensory–motor perceptions through language with special focus on natural language use by architects. The focus of much research on sensory perceptions has centred on vocabulary, naming, and codability and not on how speakers in fact use language in authentic communication to describe sensory experiences. The contribution of this article, where we review previous literature and provide a case study of communication about built space by architects, is to draw attention to the multifunctionality of word meanings in language and to point to the non-trivial linking of words and meanings in actual communication. Perception is multimodal as stated by Gibson’s (1966) example in the Introduction and this multimodality and flexibility is reflected in meaning making in language use. This is an insight that becomes evident if we go out of our way to also explore production data in the wild.

Based on a synthesis of data from decades of research on the topic, we are in a position not only to show how meaning is created through language, but also the basic characteristics of descriptions of built space by architects. We show that two main types of perceptual descriptions afforded by built space are prominent: One is the properties related to sight, sound, touch, and taste, where the multifunctionality of the sensory descriptors is found to be highly flexible in terms of meaning application. For instance, the use of words that at first blush may be considered as cues to sound in fact refer to sight (e.g., *quiet, muted,* and *discordant*). Likewise, expressions that at first may be considered expressions that refer to sound (e.g., *glare, transparency,* and *shade*) or touch are often used to describe sight (e.g., *crisp, coarse,* and *warm*). These descriptors are primarily expressing experiences of states, which is natural since built space represents something stative and stable.

However, the other characteristic of perceptual descriptions of built space is instantiated in the domain of motion, and hence the stative and stable nature of built space is portrayed in a dynamic way. In the descriptions where motion is involved, the architect may take one out of two possible positions in terms of perspective. The architect may describe built space from the point of view of an observer and built space then is described as if it were a dynamic entity. These descriptions are personifications, i.e., buildings have animate properties (e.g., verb expressions such as built space *reaches out to the natural landscape, climbs up, unfolds, runs along, hunkers, crouches,* and *wraps around,* as well as with nominal descriptions to a similar effect such as *paths, destinations, promenades, and routes*). In addition to this observer perspective, we also find descriptions where the architect is an agent moving through built space describing personal experiences while moving around (e.g., *I confront the city with my body; my legs measure the length of the arcade and the width of the square; my gaze unconsciously projects my body onto the facade of the cathedral, where it roams over the mouldings and contours*). In this case, the reader experiences the space through the bodily experiences of the architect, infusing life into the description of the feelings of motion, sight, and touch.

The language of architects is a relevant domain because it not only points to the critical role of language in the work of architects as opposed to folk ideas about architecture as an exclusively technical and/or graphic craft (consider, for instance, the many architect–client interactions involved in any design, or the reviews of notorious buildings in quality newspapers and specialized publications), but it also provides a window into the ways and the wordings that people in general use to share perceptions through language. That is, the flexibility of the use of words across modalities to evoke meanings that speakers may regard as alien to those domains, if asked out of context. To be able to explain how we share perceptual impressions through language, (i) a theoretical framework such as cognitive semantics that takes seriously the grounding of meanings in the body and the senses, (ii) a focus on authentic language, and (iii) an appreciation of the dynamics of meaning making and use of language resources are necessary components.

Through this contribution, we also want to broaden the perspective and point to the importance of research on language use in authentic communication. The expressions discussed in this article are not peculiar expressions that belong to a specific domain or profession, but expressions that are used in the same dynamic ways in human communication more broadly. This multifunctionality of words that range over several domains points to the human inclination towards cognitive ecology and the entailing dynamics of language. Knowledge of how meanings in language are cued is not only essential for researchers in the language sciences and for work related to semantic analysis but is highly relevant for researchers in other fields where natural language is used and conclusions are drawn from language data.

## Figures and Tables

**Table 1:** (Cross)sensory language.

source sense → target sense
sound → sight quiet, mute(d), grandiloquent, hushed, jarring, cacophony, cacophonous, discordant, noise, tone down, resonate sound → sight + touch rhythm, rhythmic, beat, melody, orchestrate, choreograph
sight → sound transparency, glare, shadow/shade
touch → sound abrasions touch → sight crisp touch → touch + sight coarse, coarseness, grain, warm, warmth, cold, cool, fragile, hard, soft(en), light(en), tactile, coarse, weight, heavy, sharp
taste → sight bland, insipid

## Data Availability

Data availability in this contribution is not relevant since we discuss findings reported in previous work by Caballero (2006, 2009, 2017, 2020) and (Casakin 2019).

## Notes

1 https://www.royalacademy.org.uk/exhibition/sensing-spaces (accessed on 1 January 2023).

2 Clearly, there are also meanings in language that are less sensory than the meanings we deal with here, for instance, more abstract shell concepts such us “idea”, “politics” “news” and the like.

3 Unless otherwise indicated, all the examples discussed in this chapter belong to the 150-text corpus described at the beginning of this section.

4 In *De Anima*, Aristotle offered a view of motion as a complex, holistic sense referred to as *common sensible* and encompassing physical properties indirectly perceived by more than one sense. This classical notion is revamped by contemporary anthropologists (Howes 2009, 2010) and literary scholars (Heller-Roazen 2007) claiming the existence of a holistic sense called *common sense,* which unifies and coordinates the other—acknowledged—five senses and their physical instantiations or expressions.
